# A history of cigarette smoking is associated with faster functional decline and reduction of entorhinal cortex volume in mild cognitive impairment

**DOI:** 10.18632/aging.202646

**Published:** 2021-02-12

**Authors:** Mayun Chen, Chaoming Hu, Haoru Dong, Hanhan Yan, Peiliang Wu, Alzheimer’s Disease Neuroimaging Initiative

**Affiliations:** 1 Department of Pulmonary and Critical Care Medicine, The First Affiliated Hospital of Wenzhou Medical University, Wenzhou, Zhejiang, People's Republic of China; 2 Renji College, Wenzhou Medical University, Wenzhou, Zhejiang, People's Republic of China; 3 The First Clinical College, Wenzhou Medical University, Wenzhou, Zhejiang, People's Republic of China; 4 Department of Respiratory Medicine, Ruian People’s Hospital, The Third Affiliated Hospital of Wenzhou Medical University, Wenzhou, Zhejiang, People's Republic of China

**Keywords:** smoking, mild cognitive impairment, Alzheimer's disease, entorhinal cortex, longitudinal study

## Abstract

Little is known about the longitudinal association of cigarette smoking with Alzheimer’s Disease (AD) related markers in subjects with mild cognitive impairment (MCI). In this study, we aimed to examine the effect of a history of cigarette smoking on change in global cognition, verbal memory, functional performance, hippocampal volume, entorhinal cortex volume, brain glucose metabolism, and CSF AD pathologies over time in MCI subjects. At baseline, there were 870 subjects with MCI, including 618 non-smokers (no history of smoking) and 252 smokers (any lifetime history of smoking). Linear mixed models were fitted for each outcome with adjustment of several covariates. The major findings were: (1) Among older people with MCI, smokers showed faster decline in functional performance compared to non-smokers; (2) Smokers demonstrated steeper decline in entorhinal cortex volume than non-smokers; (3) A history of cigarette smoking was not associated with change in CSF Aβ42, t-tau or p-tau levels over time in MCI subjects. In conclusion, we found that a history of cigarette smoking was associated with faster decline in functional performance and entorhinal cortex volume over time at the prodromal stage of dementia.

## INTRODUCTION

Growing evidence has shown that cigarette smoking is a potential modifiable risk factor for Alzheimer’s Disease (AD) [1]. However, the neurobiological mechanism by which cigarette smoking increases the risk of AD remains unclear. To address this question, several studies have attempted to examine the effect of cigarette smoking on AD-related markers, such as global cognition, verbal memory, hippocampal volume, brain glucose metabolism, and AD pathologies in the brain [2–8].

Mild cognitive impairment (MCI) is regarded as a prodromal state for AD dementia [9]. MCI patients have a very high conversion rate to AD dementia, with 50% to 70% progressing to dementia within the next five to seven years after a diagnosis of MCI [10]. Surprisingly, the evidence on the effect of cigarette smoking on AD-related markers in MCI is scarce. In addition, no prior studies have attempted to examine the association of cigarette smoking with change in brain glucose metabolism or CSF AD pathologies over time in MCI patients. Therefore, in the present study, we aimed to systematically investigate the association of cigarette smoking with change in global cognition, verbal memory, functional performance, hippocampal volume, entorhinal cortex volume, brain glucose metabolism, and CSF AD pathologies over time in MCI subjects. Our findings may contribute to a better understanding of the underlying mechanism by which cigarette smoking increases the risk of developing AD.

## RESULTS

### Demographical and clinical variables by smoking history

Table 1 demonstrates the demographical and clinical information by smoking history. At baseline, there were 870 subjects with MCI, including 618 non-smokers and 252 smokers. Compared to non-smokers, smokers were less likely to be females while more likely to be APOE4 carriers (p < 0.05). However, there was no difference in age or education between two groups (all p > 0.05). Smokers had lower MMSE score (lower score indicates more severe cognitive impairment) and RAVLT total score (lower score indicates more severe cognitive impairment) than non-smokers. Further, smokers showed more severe functional impairment as assesses by FAQ (higher score indicates more severe functional impairment) compared to non-smokers. In term of the difference in neuroimaging markers, smoking was associated with lower HpVR and EVR, but not FDG SUVRs. There was no significant difference in CSF AD pathologies between two group. In addition, the number of individuals with MMSE data at each follow-up visit is also presented in Table 1.

**Table 1:** Demographical and clinical variables by smoking history.

**Variables**	**Non-smokers (n = 618)**	**Smokers (n = 252)**	**P value**
Age, years	72.7 ± 7.66	73.8 ± 7.33	0.08
Education, years	16 ± 2.86	15.7 ± 2.79	0.22
Female gender, n (%)	270 (43.7)	85 (33.7)	0.007
APOE4 carriers, n (%)	296 (47.9)	142 (56.3)	0.02
MMSE	27.7 ± 1.79	27.3 ± 1.83	< 0.001
FAQ	2.94 ± 3.88	3.8 ± 4.57	0.009
RAVLT total score	34.8 ± 10.7	32.6 ± 10.6	0.002
FDG SUVRs	1.25 ± 0.14	1.23 ± 0.12	0.13
HpVR	4.52 ± 0.8	4.23 ± 0.75	< 0.001
EVR	2.31 ± 0.49	2.21 ± 0.46	0.02
CSF Aβ42, pg/ml	171 ± 52	171 ± 52	0.9
CSF total tau, pg/ml	89.6 ± 51.6	93.3 ± 63.4	0.8
CSF p-tau, pg/ml	40.2 ± 23.4	37.1 ± 21.2	0.12
**Sample size at each visit**^a^			
Baseline	618	252	
0.5	583	232	
1	573	221	
1.5	197	132	
2	485	194	
3	412	164	
4	298	108	
5	196	66	
6	153	60	
7	109	36	
8	55	24	
9	30	12	
10	24	4	
11	14	2	
12	10	1	
13	2	0	

^a^The number of individuals with MMSE data at each follow-up visit.

Abbreviations: MMSE: Mini-mental state examination; FAQ: Functional Activities Questionnaire; RAVLT: Rey Auditory Verbal Learning Test; FDG SUVRs: fluorodeoxyglucose standardized uptake values ratios; HpVR: Hippocampal volume ratio; EVR: Entorhinal cortex volume ratio; Aβ: β-amyloid; t-tau: total tau; p-tau: phosphorylated tau.

### Results of linear mixed models

**Figure 1:**
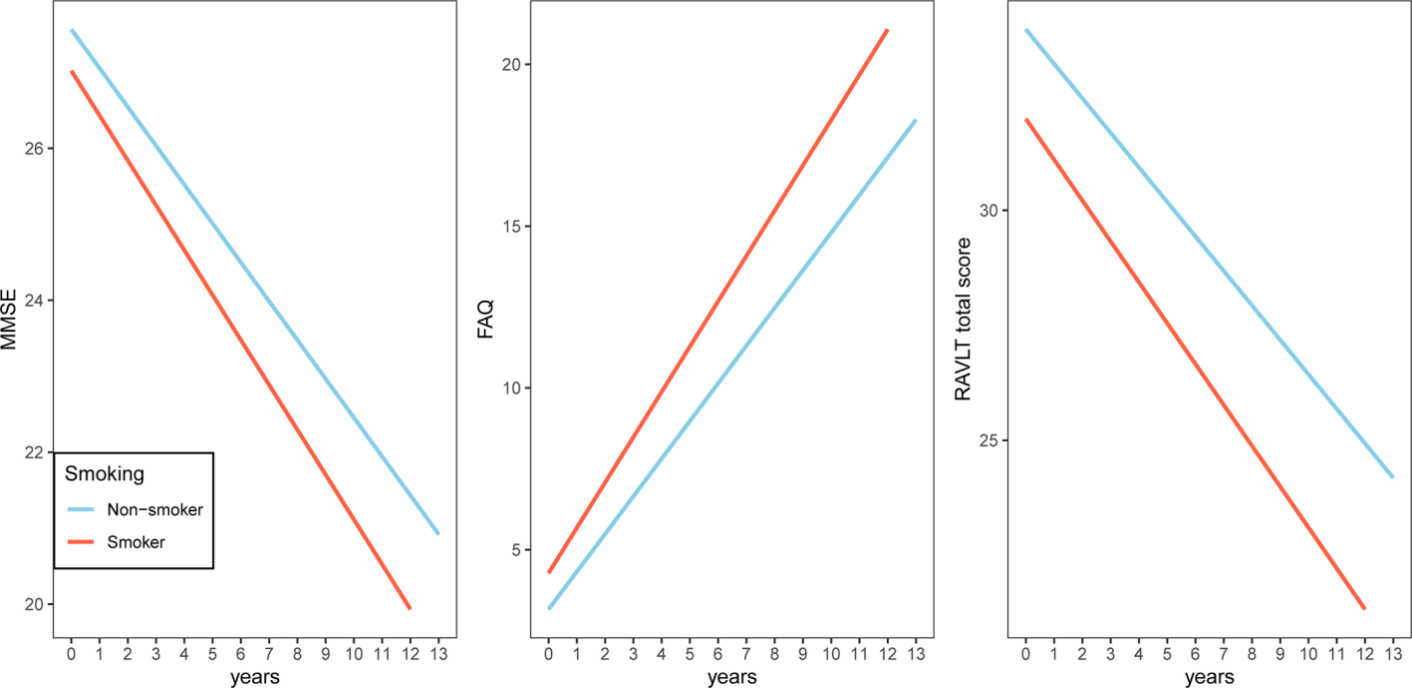
**Association of a history of cigarette smoking with change in MMSE, FAQ and RAVLT total score over time in MCI subjects.** A history of cigarette smoking was associated with change in FAQ, but not MMSE or RAVLT total score. Abbreviations: Abbreviations: MMSE: Mini-mental state examination; FAQ: Functional Activities Questionnaire; RAVLT: Rey Auditory Verbal Learning Test.

**Figure 2:**
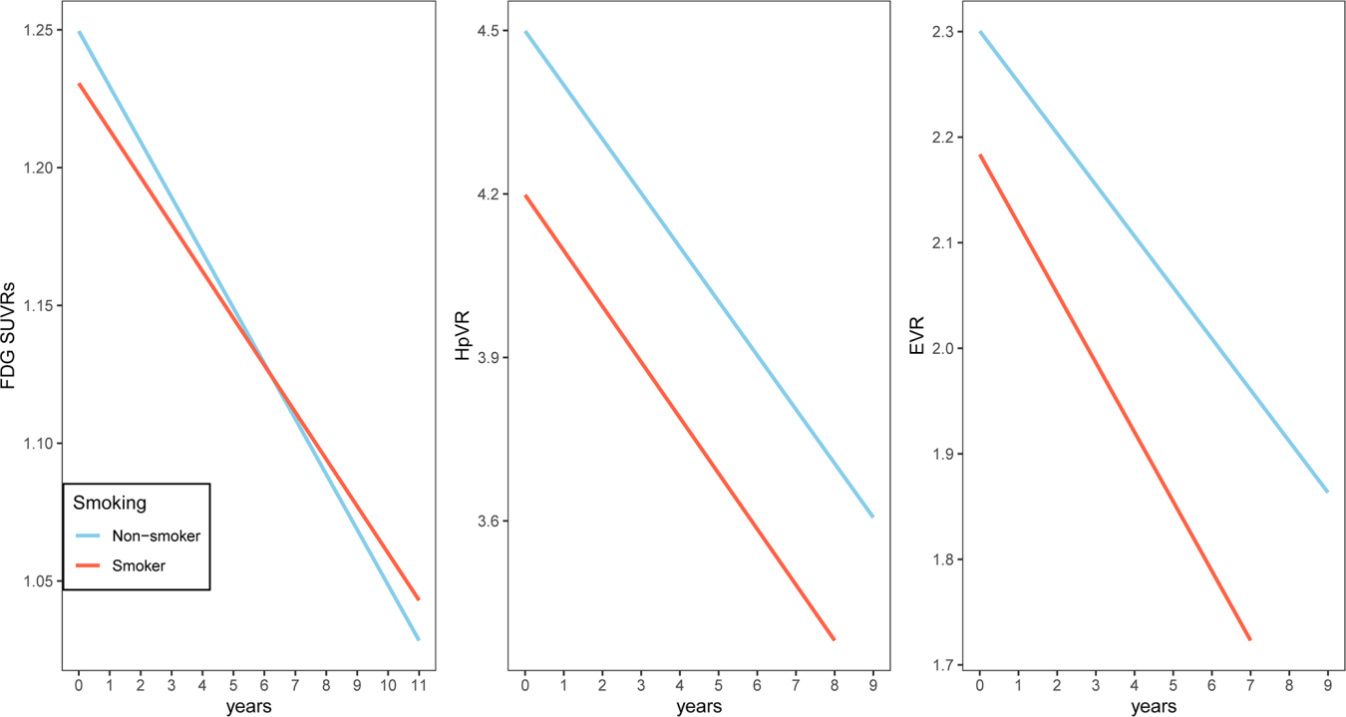
**Association of a history of cigarette smoking with change in FDG SUVRs, HpVR and EVR over time in MCI subjects.** A history of cigarette smoking was associated with change in EVR, but not FDG SUVRs or HpVR. Abbreviations: FDG SUVRs: fluorodeoxyglucose standardized uptake values ratios; HpVR: Hippocampal volume ratio; EVR: Entorhinal cortex volume ratio.

**Figure 3:**
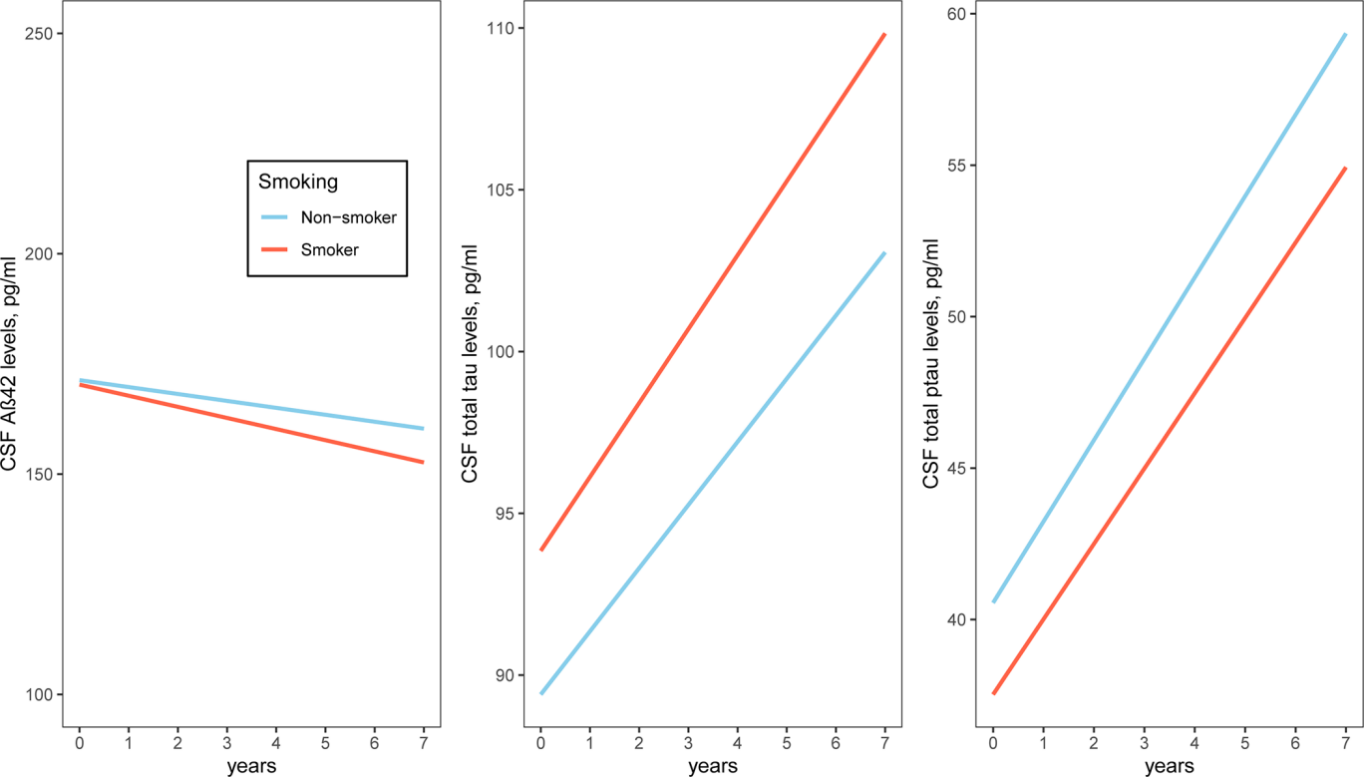
**Association of a history of cigarette smoking with change in CSF AD pathologies over time in MCI subjects.** A history of cigarette smoking was not associated with change in CSF AD pathologies. Abbreviations: Aβ: β-amyloid; t-tau: total tau; p-tau: phosphorylated tau.

To examine whether smoking was associated with changes in cognitive performance (MMSE, FAQ and RAVLT total score), neuroimaging markers (HpVR, EVR and FDG SUVRs) and CSF AD pathologies (CSF Aβ42, t-tau and p-tau levels), we fitted several linear mixed models for each outcome (Tables 2–4 and Figures 1–3). In MCI, we found that smoking was associated with changes in FAQ (estimate: 0.13, p = 0.03) and EVR (estimate: -0.02, p = 0.006), but not MMSE (estimate: -0.03, p = 0.42), RAVLT total score (estimate: 0.01, p = 0.89), HpVR (estimate: 0.0002, p = 0.96), FDG SUVRs (estimate: 0.003, p = 0.13), CSF Aβ42 (estimate: -0.72, p = 0.37), t-tau (estimate: 0.89, p = 0.4) or p-tau levels(estimate: -0.07, p = 0.93). Specifically, smoking was associated with faster decline in functional performance as assessed by FAQ (Table 2 and Figure 1). In addition, compared to non-smokers, smokers also showed steeper decline in EVR (Table 3 and Figure 2).

**Table 2:** Results of linear mixed models with cognitive and functional performance as dependent variable.

**Predictor**	**MMSE**			**FAQ**			**RAVLT total score**	
	**Estimate (SE)**	**P value**		**Estimate (SE)**	**P value**		**Estimate (SE)**	**P value**
Smoker × time	-0.03(0.04)	0.42		0.13 (0.06)	0.03		0.01 (0.09)	0.89
Age × time	-0.01(0.002)	< 0.001		0.04 (0.004)	< 0.001		-0.06 (0.006)	< 0.001
Female × time	-0.18 (0.04)	< 0.001		0.22 (0.06)	< 0.001		-0.43 (0.09)	< 0.001
APOE4 carriers × time	-0.61 (0.03)	< 0.001		1.13 (0.05)	< 0.001		-0.88 (0.08)	< 0.001
Education × time	-0.01(0.006)	0.2		0.009 (0.01)	0.38		-0.01 (0.015)	0.48

Abbreviations: MMSE: Mini-mental state examination; FAQ: Functional Activities Questionnaire; RAVLT: Rey Auditory Verbal Learning Test.

**Table 3:** Results of linear mixed models with neuroimaging markers as dependent variable.

**Predictor**	**FDG SUVRs**			**HpVR**			**EVR**	
	**Estimate (SE)**	**P value**		**Estimate (SE)**	**P value**		**Estimate (SE)**	**P value**
Smoker × time	0.003 (0.002)	0.13		0.0002 (0.004)	0.96		-0.02 (0.006)	0.006
Age × time	-0.0002 (0.0001)	0.1		-0.002 (0.0003)	< 0.001		-0.001 (0.0004)	0.002
Female × time	-0.01 (0.002)	< 0.001		-0.04 (0.004)	< 0.001		-0.02 (0.006)	< 0.001
APOE4 carriers × time	-0.01 (0.002)	< 0.001		-0.06 (0.004)	< 0.001		-0.04 (0.005)	< 0.001
Education × time	-0.001 (0.0003)	0.01		-0.0004 (0.0006)	0.54		-0.002 (0.0009)	0.006

Abbreviations: FDG SUVRs: fluorodeoxyglucose standardized uptake values ratios; HpVR: Hippocampal volume ratio; EVR: Entorhinal cortex volume ratio.

**Table 4:** Results of linear mixed models with CSF AD pathologies as dependent variable.

**Predictor**	**CSF Aβ42**			**CSF t-tau**			**CSF p-tau**	
	**Estimate (SE)**	**P value**		**Estimate (SE)**	**P value**		**Estimate (SE)**	**P value**
Smoker × time	-0.72 (0.81)	0.37		0.89 (1)	0.4		-0.07 (0.81)	0.93
Age × time	-0.02 (0.06)	0.66		-0.05 (0.07)	0.48		-0.08 (0.06)	0.17
Female × time	0.29 (0.79)	0.71		1.3 (1)	0.22		-0.57 (0.79)	0.47
APOE4 carriers × time	1 (0.74)	0.18		-0.15 (0.96)	0.87		-0.72 (0.75)	0.33
Education × time	-0.14 (0.14)	0.32		0.52 (0.18)	0.004		0.04 (0.14)	0.8

Abbreviations: Aβ: β-amyloid; t-tau: total tau; p-tau: phosphorylated tau.

## DISCUSSION

The primary findings of this longitudinal study are: (1) Among older people with MCI, smokers showed faster decline in functional performance compared to non-smokers; (2) Smokers demonstrated steeper decline in entorhinal cortex volume than non-smokers; (3) A history of cigarette smoking was not associated with change in CSF Aβ42, t-tau or p-tau levels over time at the prodromal stage of AD. Our findings may contribute to a better understanding of the underlying mechanism by which cigarette smoking increases the risk of developing AD.

The first finding that smokers had faster decline in functional performance compared to non-smokers is consistent with previous studies, which showed that smoking was associated with more impaired activities of daily living in middle-aged and older people [11, 12]. In addition, the English Longitudinal Study of Aging (ELSA) also demonstrated that among participants aged 50 or older, smoking was significantly associated with the impairment of instrumental activities of daily living [13], which is one of the key diagnostic items for dementia [14]. In the present study, we found that smoking history was associated with faster decline in functional performance in MCI subjects. To the best of our knowledge, this is the first longitudinal study to investigate the association of smoking history with change in the performance of instrumental activities of daily living at the prodromal stage of dementia.

The second finding that smokers demonstrated steeper decline in entorhinal cortex volume than non-smokers in MCI subjects is novel. In the present study, we mainly focused on two medial temporal lobe regions (hippocampus and entorhinal cortex), which are affected in the very early stage of AD [15]. However, the finding of the present study that a history of cigarette smoking was not associated with hippocampal atrophy in MCI subjects is not consistent with previous studies showing that cigarette smoking was associated with smaller volume of hippocampus in a cohort consisting of young and middle-aged participants [7]. One possible explanation for this inconsistency is that the effect of cigarette smoking on decline in hippocampus volume may be masked by the effect of AD pathologies at the prodromal stage of AD dementia.

The third finding that a history of cigarette smoking was not associated with change in CSF Aβ42, t-tau or p-tau levels over time at the prodromal stage of AD is consistent with a previously published postmortem study showing that no significant difference in levels of plaques or tangles between non-smokers and smokers was found in the midfrontal cortex of patients with AD dementia [16]. However, Tyas and colleagues observed that compared to non-smokers, smokers showed higher levels of plagues rather than tangles in the hippocampal and cerebral cortex [17]. These discrepancies may be due to several factors, such as study design (longitudinal design vs cross-sectional design), sample size and the sample (CSF vs brain tissue).

There are several potential mechanisms by which cigarette smoking affects functional decline and reduction of entorhinal cortex volume in MCI. First, chronic cigarette smoking has been found to be associated with higher risk for other cerebrovascular and cardiovascular diseases [18–21], which may contribute to the compromise of brain function and neurobiology. Second, cigarette smoking is associated with increased levels of cerebral oxidative stress [22], which may lead to neuroinflammatory responses that play a crucial role in AD pathogenesis.

Several limitations should be noted. First, compared to cognitive assessments and structural MRI data (MMSE, FAQ, RAVLT total score, HpVR and EVR), FDG and CSF AD-related biomarkers (Aβ42, t-tau, and p-tau) data had shorter follow-up duration and more missing values at each visit. Therefore, in the linear mixed models with FDG and CSF AD-related biomarkers as outcomes, the statistical power was relatively low. Future studies with larger numbers of participants with FDG and CSF AD biomarkers data at each follow-up visit or longer follow-up duration will be needed to increase the statistical power to address this issue. Second, participants of the ADNI study were highly educated and Caucasian. Thus, it may be difficult to generalize our findings to other populations. Third, given the observational nature of the present study, we cannot provide any causal evidence on the association of cigarette smoking with functional decline and reduction of entorhinal cortex volume in MCI. Future interventional studies are warranted to clarify the nature of this relationship.

In conclusion, we found that a history of cigarette smoking was associated with faster decline in functional performance and entorhinal cortex volume over time at the prodromal stage of dementia.

## MATERIALS AND METHODS

### Alzheimer’s Disease Neuroimaging Initiative

Longitudinal data were obtained from the Alzheimer’s Disease Neuroimaging Initiative (ADNI) database. The ADNI study is a longitudinal prospective study with the aim of identifying potential biomarkers of MCI and early AD (adni.loni.usc.edu). At each ADNI site across the United States and Canada, institutional review board approved the ADNI study, and informed consent was obtained from participants.

### Participants

In the present study, we included 870 subjects with MCI at baseline. MCI subjects had a Clinical Dementia Rating (CDR) [23] score of 0.5, a Mini-Mental State Examination (MMSE) [24] score of 24 or above, an objective memory decline as assessed by the Wechsler Memory Scale Logical Memory II, and no evidence of dementia. MCI subjects were further classified into two groups according to their smoking history: 618 non-smokers and 252 smokers. It should be noted that the smoker group could be further categorized into two groups: 232 former smokers and 20 active smokers. However, we combined these two groups into one due to the fact that the sample size of the active smoker group is not sufficient to conduct a longitudinal analysis.

### Cognitive assessments

The MMSE, Rey Auditory Verbal Learning Test (RAVLT) total score [25], and Functional Activities Questionnaire (FAQ) [26] were utilized to assess the global cognition, verbal memory and functional performance, respectively.

### Measurement of neuroimaging markers

The neuroimaging data, including FDG standardized uptake values ratios (SUVRs), hippocampal volume and entorhinal cortex volume, were extracted from the ADNI file “ADNIMERGE”. In the present study, to adjust for sex difference in head size, the structural MRI data were transformed based on formulas below:

Hippocampal volume ratio (HpVR) = hippocampal/intracranial volume × 10^3^

Entorhinal cortex volume ratio (EVR) = entorhinal cortex volume/intracranial volume × 10^3^

### Measurement of CSF AD pathologies

Measurement of CSF Aβ42, t-tau and p-tau levels was conducted using the multiple xMAP Luminex platform (Luminex Corp) with Innogenetics (INNO-BIA AlzBio3) immunoassay kit-based reagents, details of which could be found elsewhere [27]. All values were given as pg/ml.

### Statistical analysis

To examine the difference in continuous variables (Age, education, MMSE, FAQ, RAVLT total score, HpVR, EVR, FDG SUVRs, CSF Aβ42, t-tau and p-tau) between two smoking groups (non-smokers vs smokers), Wilcoxon test was used. To compare the frequency of categorical variables (gender and APOE4 genotype) between two smoking groups, x^2^ test was conducted. In order to examine the longitudinal relationship between smoking history and cognitive assessments, neuroimaging markers and CSF AD pathologies, several linear mixed models were performed for each outcome (MMSE, FAQ, RAVLT total score, HpVR, EVR, FDG SUVRs, CSF Aβ42, t-tau and p-tau). All linear mixed models included main effects of smoking status, age, education, gender, APOE4 genotype, and their interactions with time, as well as a random intercept for each subject.
